# Transcatheter aortic valve implantation in a patient with the left coronary artery originating directly above the annulus: images in cardiology

**DOI:** 10.1093/ehjcr/ytae212

**Published:** 2024-04-18

**Authors:** Chong Bin Lee, Hristian Hinkov, Henryk Dreger, Christoph Klein, Axel Unbehaun

**Affiliations:** Department of Cardiology, Angiology and Intensive Care Medicine, Deutsches Herzzentrum der Charité (DHZC), Augustenburger Platz, 13353, Berlin, Germany; Charité-Universitätsmedizin Berlin, corporate member of Freie Universität Berlin and Humboldt-Universität zu Berlin, Charitéplatz 1, 10117 Berlin, Germany; Charité-Universitätsmedizin Berlin, corporate member of Freie Universität Berlin and Humboldt-Universität zu Berlin, Charitéplatz 1, 10117 Berlin, Germany; Department of Cardiothoracic and Vascular Surgery, Deutsches Herzzentrum der Charité (DHZC), Berlin, Germany; DZHK (German Center for Cardiovascular Research), Partner Site Berlin, Berlin, Germany; Department of Cardiology, Angiology and Intensive Care Medicine, Deutsches Herzzentrum der Charité (DHZC), Augustenburger Platz, 13353, Berlin, Germany; Charité-Universitätsmedizin Berlin, corporate member of Freie Universität Berlin and Humboldt-Universität zu Berlin, Charitéplatz 1, 10117 Berlin, Germany; DZHK (German Center for Cardiovascular Research), Partner Site Berlin, Berlin, Germany; Department of Cardiology, Angiology and Intensive Care Medicine, Deutsches Herzzentrum der Charité (DHZC), Augustenburger Platz, 13353, Berlin, Germany; Charité-Universitätsmedizin Berlin, corporate member of Freie Universität Berlin and Humboldt-Universität zu Berlin, Charitéplatz 1, 10117 Berlin, Germany; Charité-Universitätsmedizin Berlin, corporate member of Freie Universität Berlin and Humboldt-Universität zu Berlin, Charitéplatz 1, 10117 Berlin, Germany; Department of Cardiothoracic and Vascular Surgery, Deutsches Herzzentrum der Charité (DHZC), Berlin, Germany; DZHK (German Center for Cardiovascular Research), Partner Site Berlin, Berlin, Germany

## Case description

Transcatheter aortic valve implantation (TAVI) has become a first-line treatment strategy for many patients with a calcified tricuspid aortic valve stenosis and a safe transfemoral access route.^1^ Based on computed tomography (CT) strategy planning, coronary artery obstruction today is a rare but still life-threatening complication of TAVI.^2^ In particular, a coronary ostia height of <12 mm has been identified as a significant risk factor for coronary artery occlusion.^2^

**Figure 1:**
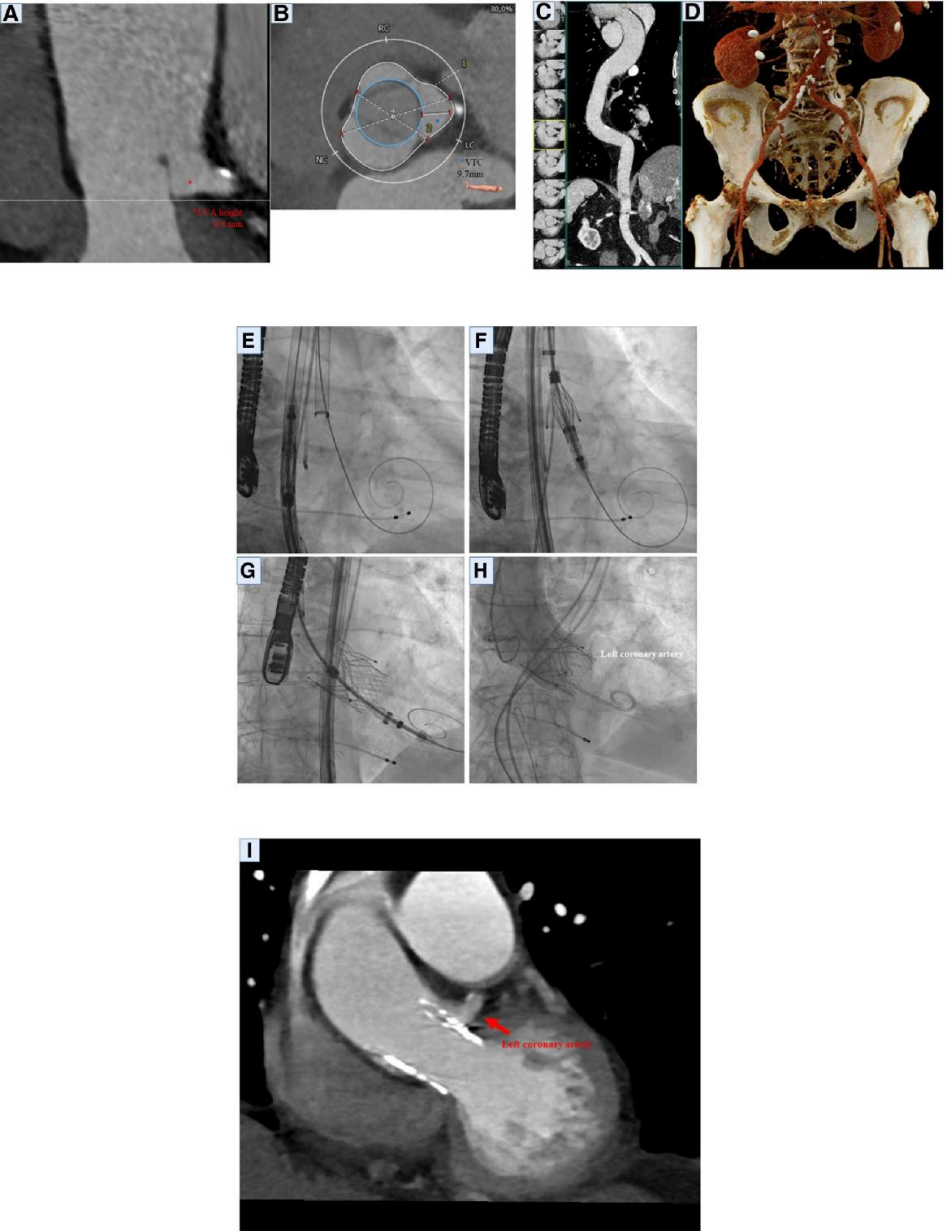
Computed tomography (CT) showing the device landing zone and the left coronary artery ostium height (*A*). Computed tomography depicting the virtual valve-to-coronary distance (*B*). Computed tomography angiography from the aortic valve to the descending aorta (*C*) and three-dimensional reconstruction of the femoral arteries (*D*). Angiography showing the valve delivery system in the ascending aorta (*E*), Trilogy valve prosthesis implantation under rapid pacing (*F* and *G*), and final angiography confirming left coronary artery perfusion (*H*). Computed tomography angiography (*I*) showing perfusion of the left coronary artery after transcatheter aortic valve implantation. LCA, left coronary artery; VTC, virtual valve-to-left coronary distance.

In this article, we present a case of a 77-year-old patient suffering from severe pure aortic regurgitation (AR) (see Supplementary material online, *Video S1*) and signs of acute heart failure. Due to the patient’s age and associated risk factors, the institutional heart team decided for TAVI. Notably, the patient’s left coronary artery (LCA) exhibited an exceedingly low take-off height of only 0.8 mm (*Figure 1A*); however, the virtual valve-to-left coronary distance was 9.7 mm (*Figure 1B*). Therefore, decision was made for TAVI with transfemoral access route (*Figure 1C* and *D*). Transcatheter aortic valve implantation utilizing the innovative JenaValve Trilogy device was performed successfully and without complications (*Figure 1E*–*H* and Supplementary material online, *Video S2*). Post-procedural analysis confirmed LCA perfusion (*Figure 1I*). At the 3-month follow-up, the patient exhibited a significant improvement in symptoms and a maintained excellent prosthesis’ function (see Supplementary material online, *Video S3*).

To our best knowledge, an LCA ostium height of 0.8 mm is the lowest reported coronary take-off height at which TAVI with the JenaValve Trilogy device was successfully performed. Obviously, the unique features of the Trilogy technology (anatomical alignment, deflecting the native leaflets away from coronary ostia by placing locators behind the cusps) allow to be more liberal in accepting those patients who present with low origins of their coronary arteries. This case underscores the pivotal role of meticulous pre-procedural imaging-driven strategy planning and exemplifies the evolving technology’s capacity to enhance the safety of TAVI, even in complex anatomical scenarios.

## Supplementary Material

ytae212_Supplementary_Data

## Data Availability

The data evaluated in this report are not available in a public repository but will be made available to other researchers upon reasonable request.
